# A novel canine model for Duchenne muscular dystrophy (DMD): single nucleotide deletion in *DMD* gene exon 20

**DOI:** 10.1186/s13395-018-0162-1

**Published:** 2018-05-29

**Authors:** Sara Mata López, James J. Hammond, Madison B. Rigsby, Cynthia J. Balog-Alvarez, Joe N. Kornegay, Peter P. Nghiem

**Affiliations:** 1 Department of Veterinary Integrative Biosciences, College of Veterinary Medicine and Biomedical Sciences, Texas A&M University, College Station, TX 77843-4458 USA; 2 Department of Neurology and Neurosurgery, Pieper Memorial Veterinary Center, Middletown, CT 06457 USA

**Keywords:** Whole genome sequencing, Next-generation sequencing, DMD, Duchenne muscular dystrophy, Dystrophin, CXMD, Animal model, Canine

## Abstract

**Background:**

Boys with Duchenne muscular dystrophy (DMD) have *DMD* gene mutations, with associated loss of the dystrophin protein and progressive muscle degeneration and weakness. Corticosteroids and palliative support are currently the best treatment options. The long-term benefits of recently approved compounds such as eteplirsen and ataluren remain to be seen. Dogs with naturally occurring dystrophinopathies show progressive disease akin to that of DMD. Accordingly, canine DMD models are useful for studies of pathogenesis and preclinical therapy development. A dystrophin-deficient, male border collie dog was evaluated at the age of 5 months for progressive muscle weakness and dysphagia.

**Case presentation:**

Dramatically increased serum creatine kinase levels (41,520 U/L; normal range 59–895 U/L) were seen on a biochemistry panel. Histopathologic changes characteristic of dystrophinopathy were seen. Dystrophin was absent in the skeletal muscle on immunofluorescence microscopy and western blot. Whole genome sequencing, polymerase chain reaction, and Sanger sequencing revealed a frameshift, single nucleotide deletion in canine *DMD* exon 20, position 27,626,466 (c.2841delT mRNA), resulting in a stop codon six nucleotides downstream. Semen was archived for future line perpetuation.

**Conclusions:**

This spontaneous canine dystrophinopathy occurred due to a novel mutation in the minor *DMD* mutation hotspot (between exons 2 through 20). Perpetuating this line could allow for preclinical testing of genetic therapies targeted to this area of the *DMD* gene.

**Electronic supplementary material:**

The online version of this article (10.1186/s13395-018-0162-1) contains supplementary material, which is available to authorized users.

## Background

Duchenne muscular dystrophy (DMD) is an X-linked, degenerative muscle disease that affects ~ 1 in 5000 males caused by *DMD* gene mutations and a resulting lack of the protein dystrophin [1]. Dystrophin anchors the sarcolemmal membrane by connecting cytoskeletal actin filaments to an associated glycoprotein complex [2]. Untreated DMD boys typically lose ambulation by 12 years of age and succumb to cardiopulmonary failure by their twenties or thirties [3]. Mutations may occur throughout the 79 exons of the *DMD* gene but concentrate in major (exons 45–53) and minor (exons 2–20) hotspot areas [4]. According to Leiden’s database [5], ~ 40% of *DMD* gene mutations are deletions of a mean size of 6.5 exons, with exon 47 being most commonly affected [4]. Duplications occur most frequently in exon 20.

There are several naturally occurring mammalian DMD models, including the X-linked muscular dystrophy mouse (mdx) [6], canine X-linked muscular dystrophy (CXMD) dogs [7–9], pigs [10], and cats [11]. Dystrophin-deficient dogs have progressive disease that largely parallels the course of DMD [8, 12]. The golden retriever (GRMD) canine model has been used most extensively for preclinical testing [13]. In GRMD, a splice site mutation in intron 6 causes deletion (skipping) of exon 7 in the *DMD* transcript, with a resulting frameshift and premature stop codon in exon 8 [14]. Several additional *DMD* mutations, including variably sized deletions and insertions, have been characterized in other dogs [13, 15, 16].

Together, studies in mammalian models have provided a better understanding of DMD pathogenesis and allowed for preclinical testing to determine both safety and potential efficacy of a range of treatments. However, with the advent of gene replacement, exon skipping, and gene editing approaches that allow treatment of specific mutations, additional large animal mammalian models with *DMD* gene mutations paralleling those of DMD are needed.

## Case presentation

A 5-month-old, male border collie dog was presented in September 2016 to a practicing veterinarian for clinical signs consistent with neuromuscular disease. The owner had obtained the dog from a breeder and did not have knowledge of his littermates or the sire and dam. He was subsequently referred to a board-certified veterinary neurologist (JJH) for further evaluation. Multiple attempts by JJH to contact the breeder for more pedigree information were unsuccessful.

**Fig. 1:**
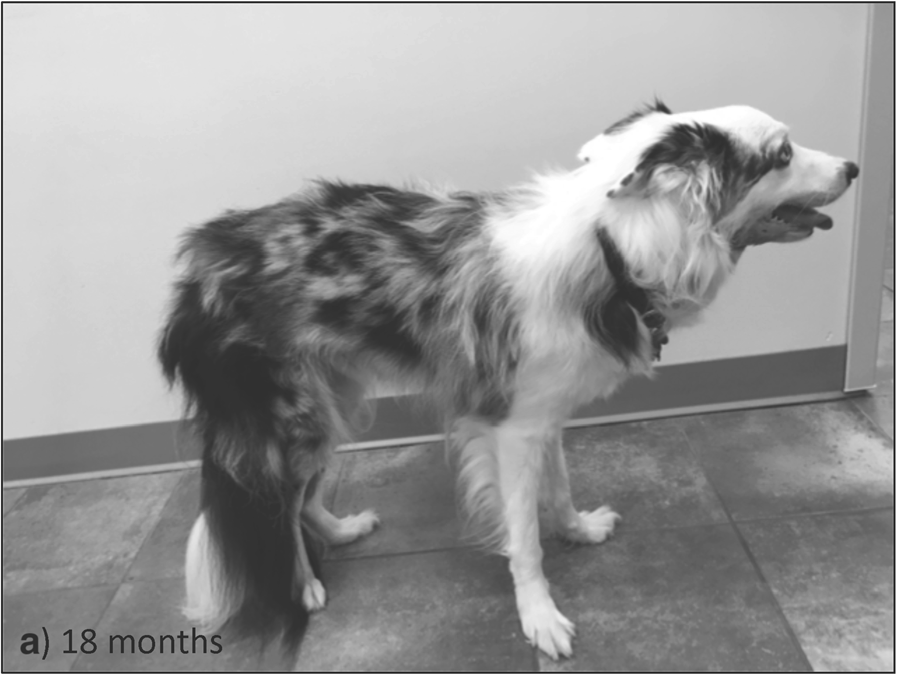
Postural changes. **a** At the age of 1.5 years, the dog had a palmigrade and plantigrade stance in all limbs and the pelvis was shifted in a cranioventral direction

On examination, fatigue and a short-strided gait were observed (Fig. 1). Postural reactions were normal when the dog’s body was supported. Muscle tone and spinal reflexes were normal, but generalized muscle atrophy was observed, most prominent in the distal limb musculature. Muscles of the proximal thoracic limbs and at the base of the tongue were prominent. Cranial nerve evaluation was normal. Drooling was reported by the owners historically and was present during the exam. Neuroanatomical localization was consistent with a generalized neuromuscular disorder.

Masticatory, lingual, paraspinal, supraspinatus, and cranial tibial (CT) muscles were examined with bipolar needle electromyography while the dog was under general anesthesia (isoflurane and oxygen). Complex repetitive discharges were detected, most pronounced in the lingual and proximal thoracic limb muscles.

At 6 months, blood was taken for biochemical and genomic analysis and surgical biopsies were performed on the vastus lateralis (VL) and CT muscles. Muscle samples were placed on a wooden tongue depressor, wrapped in a sterile saline-soaked gauze pad, and shipped overnight on a cold pack to Texas A&M University (laboratory of PPN). The samples were immediately frozen in liquid nitrogen-chilled isopentane and stored at − 80 °C.

As detailed above, no pedigree information was available on this dog. Hence, there were no carriers from which the condition could be perpetuated through breeding. With this in mind, semen from the dog was collected at a recheck appointment and frozen in liquid nitrogen for future line perpetuation. By inseminating a normal dog, all female progeny would be obligate carriers.

Blood chemistry results showed elevated levels of AST, ALT, BUN/creatinine ratio, phosphorus, glucose, and notably, creatine kinase (CK 41,520 U/L; Table 1). Platelets were mildly elevated. Remaining blood count and chemistry values were within normal range. No antibodies were detected against *Toxoplasma gondii* or *Neospora caninum*.

**Table 1:** Blood chemistry results for the affected border collie showed muscle-specific changes

Lab finding	Values	Normal range
AST (SGOT) (U/L)	671	15–66
ALT (SGPT) (U/L)	446	12–118
Creatinine (mg/dL)	0.4	0.5–1.6
BUN/creatinine ratio	40	4–27
Phosphorus (mg/dL)	7.6	2.5–6.0
Glucose (mg/dL)	149	70–138
Creatine kinase (U/L)	41,520	59–895
Platelet count (10^3^/μL)	489	170–400

**Fig. 2:**
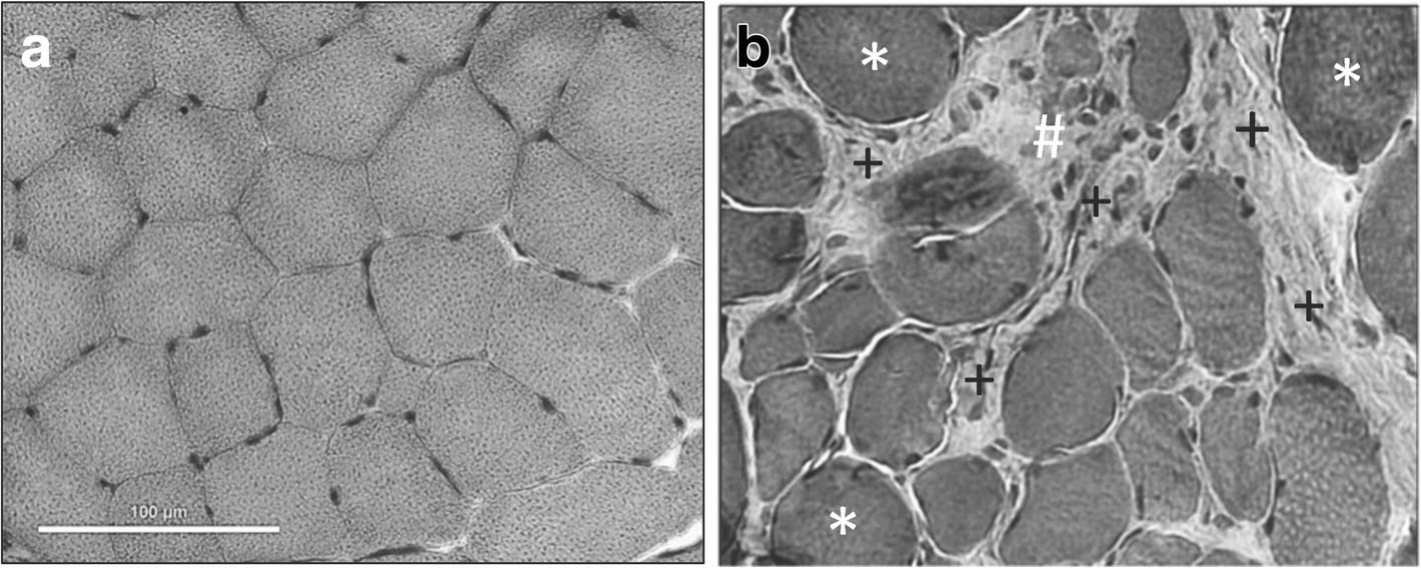
Histopathological changes consistent with dystrophinopathy. **a** Normal dog at 6 months of age showing uniform fiber size and minimal endomysial connective tissue. **b** Affected border collie vastus lateralis muscle with dystrophic changes, including myofiber size variation owing partly to larger hyaline fibers (*), increased cellularity likely due to combined effects of inflammation and satellite cell activation (#), and increased connective tissue (+). Hematoxylin and eosin (H&E). Metric bar = 100 μm in both

Tissue cryosections of the VL and CT (not shown) muscles were stained with hematoxylin and eosin (H&E) [17] and analyzed by light microscopy. There was myofiber size variation, hyaline myofiber necrosis, increased primarily endomysial connective tissue, and increased mononuclear cells likely representing a mix of inflammatory cells and activated satellite cells (Fig. 2b) (see further below).

**Fig. 3:**
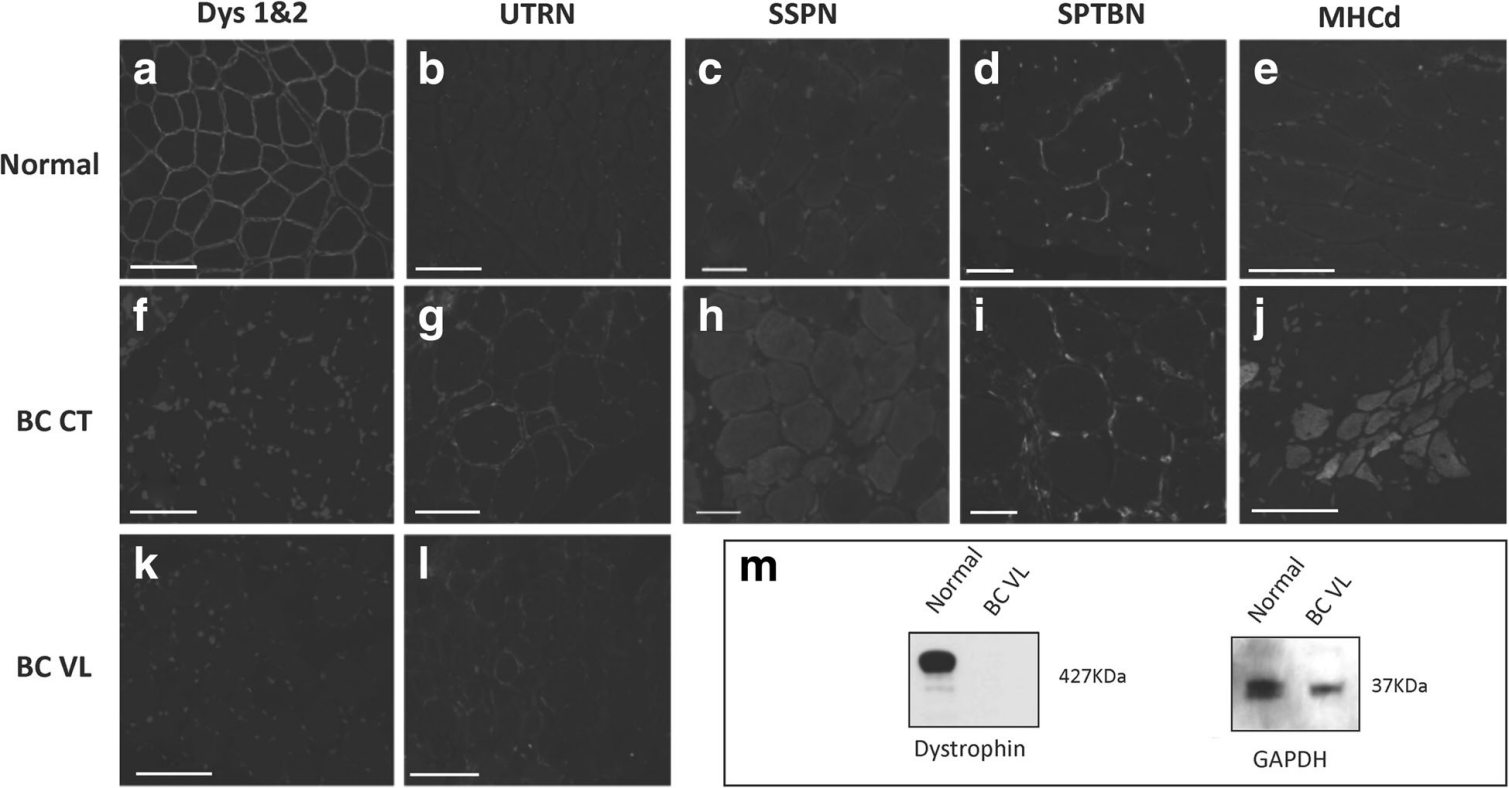
Dystrophin deficiency in the affected border collie (BC) dog. Normal and dystrophic muscle were immunostained for DYS1 and 2 (**a**, **f**, **k**). Peri-membranous dystrophin expression was seen in each myofiber of normal muscle (**a**) but was absent in the affected dog (**f**, **k**). Utrophin (UTRN) was minimally expressed in normal muscle (**b**) but, by comparison, was increased in the affected dog (**g**, **l**). Similarly, sarcospan (SSPN) was minimally expressed in normal muscle (**c**) and comparably increased in the affected dog (**h**). Spectrin (SPTBN) was used as a cellular membrane marker (**d**, **i**). Myosin heavy chain developmental fibers (MHCd) positive myofibers were absent in normal muscle (**e**) but present in the affected dog (**j**). Nuclei were stained with DAPI. All images were taken with a × 20 objective. **m** Western blot showed absent dystrophin in the BC; GAPDH was used as a loading control. Metric bar = 100 μm

Immunofluorescence microscopy was performed on VL and CT samples. Cryosections co-stained using dystrophin rod (NCL-Dys 1 Leica) and C-terminus (NCL-Dys 2 Leica) domain antibodies at 1:100 dilution and goat anti-mouse Alexa Fluor 488 secondary antibody (Life Technologies) at a 1:500 dilution were analyzed. Utrophin was stained with a primary antibody (Developmental Studies Hybridoma Bank) at 3.5 μg/mL with the aforementioned secondary antibody. Dystrophin protein was absent on immunofluorescence microscopy (Fig. 3a, f, k) compared with a normal sample. Revertant fibers were not observed. Utrophin staining was positive in some fibers with central nuclei in the border collie, but almost undetectable in normal canine tissue (Fig. 3b, g, l). Cryosections from the affected dog stained for sarcospan with a primary antibody (Origene) at 1:250 and Alexa 488 goat anti-rabbit (Life Technologies) at 1:500 (Fig. 3c, g) showed increased expression, probably associated with utrophin upregulation [18]. Spectrin (Abcam) at 1:100 as together with the same secondary mentioned above (Fig. 3d, i) was used as a cellular membrane control. Multiple inflammatory cell markers were assessed with immunofluorescence, and no definite positive cells were seen. Some myofibers in the dystrophic dog stained positive for myosin heavy chain developmental fibers (MHCd) antibody (Leica) (Fig. 3e, j) at 1:100 and 1:500 Alexa 488 goat anti-mouse (Life Technologies) antibody, consistent with satellite cell activation. All slides were co-stained with DAPI (Invitrogen) at 1:2000.

Western blotting methods have been described previously [19]. NCL-Dys 1 NCL-Dys 2 antibodies at 1:200 dilution and goat anti-mouse IgG HRP (ABCam) were incubated at a 1:5000 dilution. GAPDH was used as a loading control (Santa Cruz Technologies) after stripping the membrane (Thermo Fisher). Dystrophin protein was absent on immunostaining analysis of muscle lysates (Fig. 3m).

Genomic DNA was extracted from the blood using a Qiagen DNA extraction kit (QIAamp DNA Blood Mini Kit, QIAGEN) following methods provided by the manufacturer. Subsequent molecular characterization of the underlying *DMD* gene mutation was performed using whole genome sequencing (WGS) with methods previously described [19]. National Center for Biotechnology Information’s (NCBI) Genome Workbench software was used for data analysis. Single nucleotide polymorphisms (SNPs), deletions, and insertions in the *DMD* gene were compared to the CanFam3.1 whole genome shotgun sequence [20]. Subsequent analysis of this dog’s deleted base pair (bp) was performed with the Leiden DMD database [5].

For comparison purposes, the reference genome length for the canine *DMD* gene is 2,392,715,236 (NCBI CanFam3.1) [20] with mapped reads for the affected border collie at 2,386,159,041 (99.73% of reference genome). There was a mean depth read of 31X. The total number of reads mapped to the reference genome (608,164,144) was 573,083,874 (94.23%). There were 6,072,297 (1% of reference genome) variants composed of 612,599 deletions (10% of variants), 655,520 insertions (11% of variants), and 4,804,178 SNPs (79% of variants). The overall genomic GC content was 41.75%. There were 2531 variants within the *DMD* gene (X chromosome, NC_006621.3; 26,290,903…28,444,730 NCBI), which was relatively higher than previously reported in a Cavalier King Charles spaniel dog with WGS [19].

**Fig. 4:**
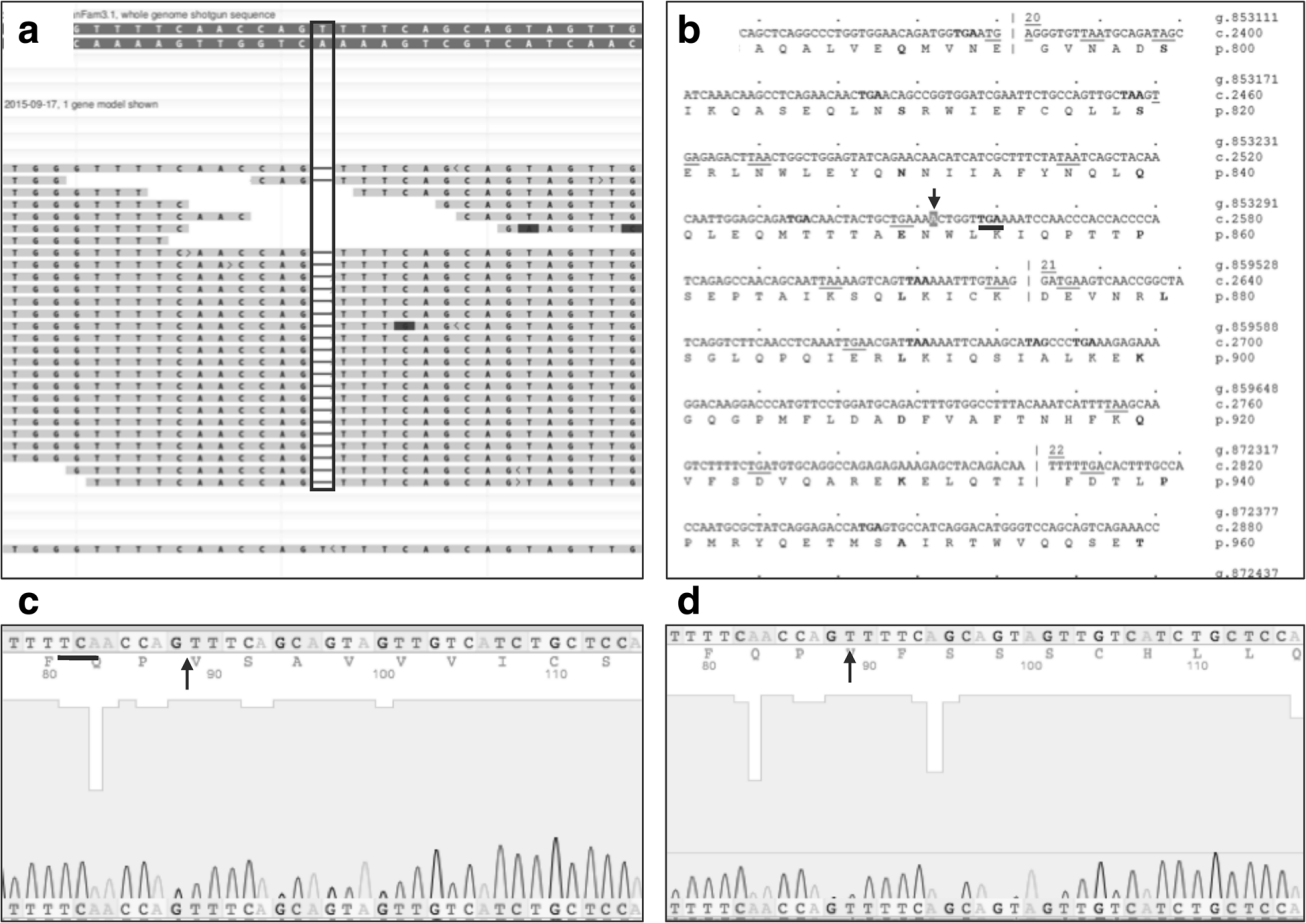
Whole genome sequencing revealed a point mutation (1 base pair deletion) in exon 20 of the canine *DMD* gene. **a** Screen shot of NCBI Genome Workbench revealing 22 reads with the point mutation (nucleotide A; black rectangle). **b** Screen shot of Leiden DMD database with the deleted nucleotide highlighted in blue (red arrow). A stop codon (TGA) present six nucleotides downstream in exon 20 (red line). **c** Sanger sequencing screen shot of the mutated area (black arrow) with the reverse strain ACT stop codon six nucleotides downstream (black line). **d** Sanger sequencing screen shot of a normal dog in the same area. Black arrow points at the normal (non-mutated) sequence

WGS revealed a 1-bp nucleotide (T) deletion in position 27,626,466 (c.2841delT mRNA) in exon 20 of the canine *DMD* gene (Fig. 4a), corresponding to position 36,636,833 (c.2552delT mRNA) in exon 20 of the human *DMD* gene. According to the Leiden DMD database [5], this nucleotide deletion would result in a stop codon six nucleotides downstream from the deletion site (Fig. 4b).

Polymerase chain reaction (PCR) and Sanger sequencing confirmed the single nucleotide deletion when compared to a normal dog (Fig. 4c, d). Outside and inside forward and reverse primers were designed to encompass the genomic DNA region containing the deletion identified by WGS (Additional file 1 Table S1). Primary PCR was performed using the outside primers and TaKaRa Ex Taq Polymerase Kit under the following conditions: 94 °C for 1 min; 94 °C for 30 s, 48.4 °C for 30 s, 72 °C for 1 min (30 times); and 75 °C for 5 min. The product of this reaction was used for secondary PCR with inside forward and reverse primers designed (Additional file 1 Table S1). The T7 sequence (TAATACGACTCACTATAG) was included on the 5′ end of the inside forward primer for Sanger sequencing. Secondary PCR was performed under the following conditions: 94 °C for 1 min; 94 °C for 30 s; 45.3 °C for 30 s, 72 °C for 1 min (30 times); and 75 °C for 5 min. Gel electrophoresis (1.3% agarose) was used to determine the quality of PCR products. The desired band (223 bp) was excised from the gel and the DNA purified (QIAEX II Gel Extraction Kit Qiagen). Purified secondary PCR product was submitted for Sanger sequencing (Eton Bioscience; Texas A&M University).

In addition to this novel deletion in *DMD* exon 20, two additional non-synonymous substitutions were identified in *DMD* exons 15 (position 27,697,781; serine AGC to asparagine AAC) and 34 (position 27,512,289; alanine GCG to serine TGC). Using the Ensembl database [21], there was also a T deletion at position 26,290,826 in the untranslated region of exon 79. In contrast, when the NCBI database was used, this deletion fell outside the untranslated region of exon 79. Finally, the previously published GRMD “escaper” single nucleotide substitution in the gene *Jag1* [22] was not present.

## Discussion and conclusions

This study describes a novel *DMD* gene mutation in a border collie dog that could potentially be a valuable preclinical model. While this dog had several *DMD* gene mutations, we believe the T nucleotide deletion in exon 20 most likely led to the loss of dystrophin. Located in the exon 2–20 minor hotspot for the *DMD* gene [5], this mutation would result in a stop codon 6 bp downstream [23]. The other mutations in exons 15 and 34 were non-synonymous substitutions, expected to change the amino acid but not disrupt the reading frame. Exon 20 is most frequently duplicated in both Becker’s muscular dystrophy and DMD but can also be deleted with other exons. In Leiden’s database, exon 20 deletions have been reported in 27 cases of both DMD and Becker patients, having an incidence of 0.08% (total of 2432 BMD/DMD patients). Notably, even though this dog was alive at 22 months and had a relatively mild phenotype, it did not have the “escaper mutation” in the *Jag1* gene described by Vieira et al. [22]. Our laboratory has recently published on a large cohort of variably affected GRMD dogs without the *Jag1* mutation [24].

Blood values and histopathological changes in this border collie were consistent with those of other dystrophin-deficient dogs [7, 25]. He was seen in the clinic at 5 months with signs of muscle atrophy, macroglossia, fatigue during ambulation, drooling, and “bunny hopping” gait. At the time of this study, the dog continued to live with his owners. His clinical signs had largely stabilized, in keeping with mildly affected dystrophic dogs seen in our own lab [7, 13, 24] and by others [8, 9, 19, 25].

Due to the range of *DMD* gene mutations observed in patients, this new potential canine model could be useful in testing genetic therapies. Novel exon skipping compounds, whereby exons 19 and 20 or 20 and 21 are skipped to restore the reading frame, could potentially be tested. In addition, techniques such as TALEN or CRISPR/Cas9 could be utilized for single bp restoration (i.e., homology directed repair) or exon "snipping" (i.e. non-homologous end joining). With this in mind, the semen was collected to allow perpetuation of the line myoblasts which were extracted for future immortalization.

Over and above the potential preclinical value of this new model, our work further demonstrates the value of WGS as a tool to characterize canine *DMD* gene mutations [19]. Whole genome and exome sequencing provide valuable techniques to detect mutations ranging from a single bp to multi-exon deletions. We have previously utilized WGS to identify a 7-base pair mutation in *DMD* exon 42 of a Cavalier King Charles spaniel (CKCS) dog [19], distinct from the splice site mutation reported earlier by Walmsley et al. [8]. A recent study utilized whole exome sequencing to identify two distinct mutations in the *sarcoglycan-δ* (SGCD) gene of Boston terrier dogs with a condition akin to limb girdle muscular dystrophy of humans [26].

In conclusion, WGS was used to characterize a novel single bp deletion in exon 20 of the canine *DMD* gene. These studies provide another example of the power of next-generation sequencing technology in the diagnosis of genetic animal diseases. If perpetuated, this condition could serve as a valuable model for testing genetic therapies.

## Additional file

Additional file 1: **Table S1.** Nested PCR primers. (DOCX 14 kb)

## Abbreviations

Bp: Base pair
CKCS: Cavalier King Charles Spaniel
CT: Cranial tibialis
CXMD: Canine X-linked muscular dystrophy
DMD: Duchenne muscular dystrophy
GDNA: Genomic DNA
GRMD: Golden retriever muscular dystrophy
H&E: Hematoxylin and eosin
Mdx: Murine X-linked muscular dystrophy
MHCd: Myosin heavy chain developmental
NCBI: National Center for Biotechnology Information
PCR: Polymerase chain reaction
SGCD: Sarcoglycan-δ
SNPs: Single nucleotide polymorphism
VL: Vastus lateralis
WES: Whole exome sequencing
WGS: Whole genome sequencing
