# Supplementary material for: A novel canine model for Duchenne muscular dystrophy (DMD): single nucleotide deletion in DMD gene exon 20
Source: Skelet Muscle. 2018 May 29;8:16. doi: 10.1186/s13395-018-0162-1 (PMC5975675; doi:10.1186/s13395-018-0162-1)
Supplement: Supplementary file 1 — Table S1. Nested PCR primers. (DOCX 14 kb) [file 13395_2018_162_MOESM1_ESM.docx]

**Additional files**

**Supplemental Table 1: Nested PCR primers**

| **Primary PCR – Outside Primers** | |
| --- | --- |
| *Forward* | *Reverse* |
| 5’-AGGATCCAGACATCATGCA-3’ | 5’-GCTGTCAGCATTAACACCCT-3’ |
| **Secondary PCR – Inside Primers** | |
| *Forward* | *Reverse* |
| 5’-GATCATGATCCAAATAGGAGG-3’ | 5’-TTGGCAGAACTCTATCCAC-3’ |
